# Multisource working condition recognition via nonlinear kernel learning and *p*-Laplacian manifold learning

**DOI:** 10.1016/j.heliyon.2024.e26436

**Published:** 2024-02-20

**Authors:** Bin Zhou, Rui Niu, Shuo Yang, Jianguo Yang, Weiwei Zhao

**Affiliations:** a School of Computer Science and Technology, Shandong University of Technology, Zibo, China; b College of Geosciences and Engineering, North China University of Water Resources and Electric Power, Zhengzhou, China; c School of Mechanical Engineering, Shandong University of Technology, Zibo, China

**Keywords:** Multisource working condition recognition, Nonlinear kernel learning, *P*-Laplacian manifold learning, Measured signal feature, Sucker-rod pumping energy system

## Abstract

Effectively utilizing information from multiple sources and fewer labeled operating condition samples from a sucker-rod pumping system for oil production can improve the recognition effects and engineering practicability. Nevertheless, this is a challenging energy environment scientific application research subject, and therefore, this study proposes an operating state recognition scheme that relies on multisource nonlinear kernel learning and *p*-Laplacian high-order manifold regularization logistic regress. Specifically, three measured features are selected and extracted, i.e., wellhead temperature signal, electrical power signal, and ground dynamometer cards, based on mechanism analysis, expert experience, and prior knowledge. Finally, we establish the operating condition recognition model to recognize by the multisource *p*-Laplacian regularization kernel logistic regress algorithm. The experimental data are derived from 60 wells of a common high-pressure and low-permeability thin oil reservoir block of an oil field in China. The corresponding trials highlight that our scheme outperforms traditional recognition methods by exploiting single-source and multiple-feature data. In the context of fewer labeled samples, the proposed method has a greater recognition effect, engineering practicability, and better model robustness than the existing schemes based on other high-order manifold learning, verifying our method's effectiveness.

## 1 Introduction

Recognizing the operating state of a sucker-rod pumping system in time and accurately, while based on a few labeled measured data combined with multi-source data and massive unlabeled measured data, is an active research field in big data extraction and analysis of oil extraction production. In cross-research involving the information and oil extraction engineering domains, a major challenge is further improving the identification effect, model robustness, and engineering practicability of existing working condition recognition methods by utilizing multiple information sources and few known operating condition resources.

Nevertheless, the current operating condition recognition research suffers from the following constraints. First, most methods solely rely on one information source [[1], [2], [3], [4], [5], [6]], e.g., electrical parameters or dynamometer cards. However, employing one information source easily triggers a complex electron machinery liquid integrating system, producing false alarms. Second, the existing methods' model robustness and recognition performance using multi-source information must be further improved [7,8]. This is because traditional multi-feature connection methods are technically limited from being further improved. Moreover, such methods are affected by the unreliable data collected initially, e.g., statistics and production data, affecting the existing multi-source recognition methods’ stability that must be further strengthened. Third, due to the numerous labeled training samples, the engineering practicability in most operating condition recognition methods must be enhanced [9,10]. Fourth, most feature extraction schemes relying on a single information source involve several complicated calculations [11,12], which is not conducive to engineering applications. Fifth, the electric power cards and pump dynamometer cards are obtained by model transformation. However, due to the “division by zero" and damping coefficient, existing recognition algorithms based on two transformation models suffer from calculation accuracy errors about the feature parameter eigenvalue [13].

The ongoing IoT (Internet of Things) and big data era can measure large amounts of multi-source data, such as wellhead pressure and temperature, massive unknown operating condition data, electrical parameters, and ground dynamometer cards, which are extracted and stored from a sucker-rod pumping oil production system. How to effectively use these multi-source measured data to address the above limitations and further improve the accuracy, robustness and practicability of the working condition recognition methods in sucker-rod pumping system? Research has shown that multiple nonlinear kernel learning operation can effectively solve the traditional object multi-source problem, and each feature information source can learn and complement the other, improving the algorithm's performance [[14], [15], [16]]. Meanwhile, the multi-source feature-based learning methods involving high-order manifold improve the recognition effect further [17,18]. Moreover, semi-supervised learning (SSL) schemes based on *p*-Laplacian manifold regularization and loss function significantly enhance the classification performance by utilizing richer unlabeled data efficiently in fewer labeled data [19,20]. Other studies have shown that in practical engineering, data feature analysis combined with expert knowledge and mechanism analysis contributes to more efficient and practical model building [21]. In summary, combined with the actual operation of the oil well, a working condition recognition method based on measured multisource nonlinear kernel learning and *p*-Laplacian manifold learning is proposed in this paper.

This paper's contributions include three aspects: (1) implement the operating condition recognition effects to improve the performance further by employing a multiple nonlinear kernel learning algorithm relying on multiple measured information sources from a sucker-rod pumping production system; (2) introducing a *p*-Laplacian high-order manifold regularization and a log loss function into the multi-source kernel SSL to enhance the robustness and the engineering practicability further while relying on fewer labeled and massive unlabeled samples; (3) employing prior knowledge-based feature extraction, expert experience, and mechanism analysis to further enhance the model's effectiveness by calculating the feature parameter eigenvalue rapidly, accurately, and expressly.

In this research work, we focus on fusing different information sources effectively to obtain better operating condition recognition effects in the case of limited labeled samples and massive unlabeled samples from sucker-rod pumping system. Specifically, Section 2 is the research background of the proposed method in this paper, mainly focuses on two aspects: introduction to the working principle, review and summary of working condition recognition method research status. Section 3 details the proposed working condition recognition method based on multiple nonlinear kernel SSL learning and *p*-Laplacian regularization combining with the log loss function. Section 4 describes the modeling process of the working condition recognition based on the proposed method, mainly includes the selection and extraction of feature data, the concrete process of recognition modeling. To verify the effectiveness of the proposed method and model, the experiments are carried out and the results are analyzed in Section 5. Finally, the conclusions of this paper are given in Section 6.

## 2 Research background of the proposed method

In this section, the working principle of sucker-rod pumping architecture is briefly introduced, and the current research status of working condition recognition methods in sucker-rod pumping system is also comprehensively reviewed and summarized.

### 2.1 Working principle of sucker-rod pumping architecture

**Fig. 1:**
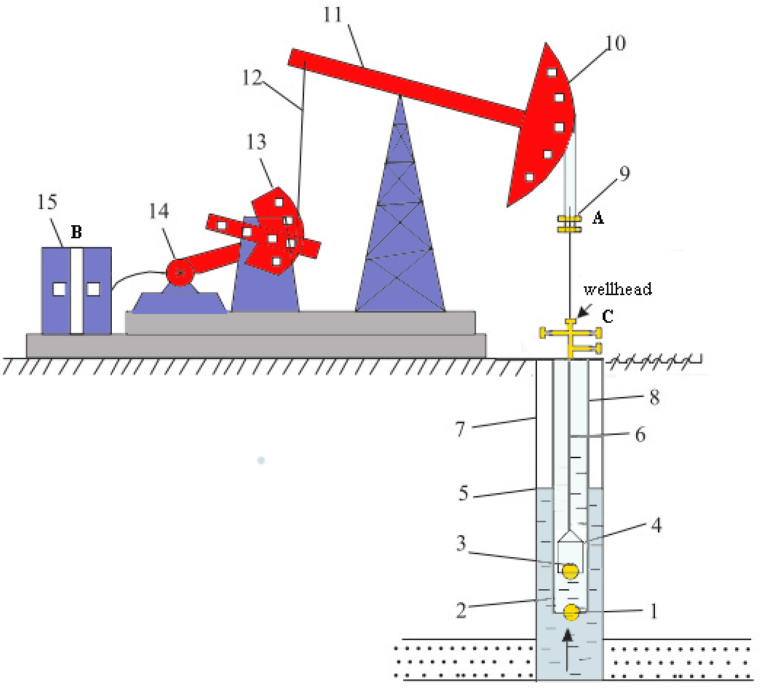
Sucker-rod pumping architecture.

As shown in Fig. 1 [11], the structure of the sucker-rod pumping architecture mainly consists of fifteen parts: (1) Standing valve, (2) Pump barrel, (3) Traveling valve, (4) plunger, (5) Dynamic fluid level, (6) Sucker rod, (7) Casing, (8) Tubing, (9) Beam hanger, (10) Horse head, (11) Beam, (12) Link rod, (13) Crank, (14) Induction motor, (15) Electronic control cabinet.

The working principle of the sucker-rod pumping architecture is as follows: after the induction motor is started, its high-speed rotating motion is transformed into the mechanical up and down swing of the beam by the crank connecting the link rod mechanism. In this transformation process, the sucker rod connected to the beam hanger suspended from the horse head drives the pump plunger in the underground wellbore to move up and down through the mechanical swing of the beam, and eventually the fluid in the wellbore is pumped to the surface.

In the sucker-rod pumping architecture, the oil well pump is the key downhole production equipment, including the pump barrel, plunger, and valves. The operating concept of the oil well pump originates from an opening and closing process of valves during a stroke.

Measured information sources in sucker-rod pumping system mainly derive from the following aspects: Dynamometer card information source (Fig. 1A, high dimensional binary image), Electronic parameter information source (Fig. 1B), Wellhead information source (wellhead temperature, wellhead pressure, etc., Fig. 1C). In the production process, the above measured data and some statistical production data, such as dynamic fluid level (Fig. 1 (5)) and liquid-producing capacity, can be regarded as information sources. The research on recognizing the operating conditions in the system usually involves effectively utilizing the above information sources.

### 2.2 Research status of working condition recognition methods

The research on the working condition recognition of sucker-rod pumping system is mainly carried out around different technology means. In view of technology means, the working condition recognition methods can be mainly divided into three categories: (1) dynamometer card-based, (2) electronic parameter-based, and (3) multi-source-based.

(1) **Dynamometer card-based recognition methods.** The dynamometer card-based identification scheme is commonly used by coupling pump or measured ground dynamometer cards with artificial intelligence. For instance, Li et al. [22] extracted the feature parameters of the pump dynamometer card (transformed by measured dynamometer cards and affected the feature parameter value calculation accuracy by damping coefficient) using the moment feature method and the “four-point method” in oil field production. Then, the authors recognized the operating state of the sucker-rod pumping system through multi-classification SVM combined with particle swarm optimization. Zheng et al. [23] obtained feature data of the ground dynamometer card with the moment feature method determined by the astr polygon decomposition centroid localization algorithm. Then, they conducted recognition using the hidden Markov model, which is optimized through a clonal selection algorithm. Zhang et al. [24] established the operating condition recognition model utilizing a sparse multi-graph regularized extreme-learning machine based on the fast discrete curvelet transform feature extraction method. Li et al. [1] proposed an improved deep learning method based on the fusion of Fourier descriptor features and graphic features of dynamometer cards. To solve the problem that the recognition effect and generalization performance of the deep learning working condition recognition model were prone to decline in few-shot training samples, He et al. [25] proposed to compress the original working condition data by using the 4-dimensional time-frequency feature extraction method, and then adjust the parameters of the convolutional shrinkage neural network by meta-learning. Overall, most of the existing dynamometer card-based recognition strategies rely on labeled samples, and most feature extraction methods utilizing single binary images involve huge and complicated calculations.
(2) **Electronic parameter-based recognition methods.** The electronic parameters mainly have two advantages: low cost and high reliability in data acquisition. Currently, three recognition method classes exist that rely on electronic parameters. Since the dynamometer card-based methods derived from electric parameter inversion do not present an appealing performance, Zheng et al. [26] extracted feature data from the measured electrical power signal through mechanism analysis and accomplished operating condition recognition using a hidden Markov model. Moreover, Chen et al. [27] deduced the transformation model of electric power cards by considering the actual angular velocity of the crank and the inertia and friction of the 4-bar linkage. Then they established a feature atlas of electric power cards to diagnose faults. Nevertheless, due to “division by zero", the recognition effects based on electronic parameters must be improved further.
(3) **Multi-source-based recognition methods.** In a complex nonlinear system, a misjudged diagnosis of the working condition can easily be made when using a single angle. Therefore, based on mechanism analysis in a sucker-rod pumping system, Zheng et al. [28] drew out seven key parameters, such as the moisture content, gas-liquid ratio, sucker-rod specifications, stroke, speed, pump diameter, and working condition, and identified the working condition using a hidden Markov model. Zhang et al. [7] selected feature data, such as dynamometer cards, power, liquid-producing capacity, dynamic fluid level. Liu et al. [8] extracted the key parameters, such as dynamometer cards, liquid-producing capacity, work day time. Besides. In the above feature data, the liquid-producing capacity, dynamic fluid level, the moisture content, and work day time are statistics, which, when influenced by uncertain statistics, the recognition effects and the model robustness based on multi-source recognition methods are not ideal.

Although the application of working condition recognition methods in sucker-rod pumping system is successful, there is still a common issue, i.e., there are few researches on the general working condition recognition method and model, which is integrated by high efficiency, robustness and practicability in a complex electron machinery liquid integrating nonlinear system. Specifically, the traditional recognition technology based on single information source or multi-feature connection or transformation model (such as pump dynamometer cards, electric power cards) affects the high efficiency, the need for labeled samples or the feature extraction technology with complex and fathomless computing affects the practicality; statistic production feature data or sample data with drastic distribution fluctuation affect the robustness. Many scholars have combined deep learning and intelligent algorithms to do a lot of research on multi-source fusion technology [16,[29], [30], [31]] and small-sample learning technology [32,33]. Compared to shallow learning techniques, deep learning techniques can learn more useful features by flexibly and efficiently building machine learning models with many hidden layers and then improve model performance. However, deep learning is costly in terms of large amounts of hyperparameter debugging, computing power cost and data quality. In addition, in the context of big data, the effective use of massive unlabeled samples can further promote the practicability of the method or model. Therefore, the merits of the proposed method in sucker-rod pumping system can be summarized as follows:

(1) The proposed method and model are an intelligent information processing method and model integrating multiple measured information sources kernel learning in a semi-supervised scene. The proposed method and model are suitable for the efficient, robust and practical complex nonlinear system.
(2) By higher-order manifold regularization, the sample data keeps manifold structure and the proposed method and model have excellent performance in the right amount of labeled samples and many unlabeled samples.
(3) Feature data is selected and extracted by expert experience, prior knowledge and mechanism analysis, and the proposed method and model can be more practical and interpretable.

## 3 The proposed recognition method based on multiple kernel SSL and *p*-Laplacian regularization

In this section, we first briefly state the effect of introducing the higher-order manifold *p*-Laplacian regularization term and the effect of the key parameter *p*-value on the graph *p*-Laplacian in *p*-Laplacian regularization, then describe in detail the derivation and solving process of the proposed recognition method based on multiple kernel SSL and *p*-Laplacian regularization, and finally analyze the complexity of the algorithm.

### 3.1 *p*-Laplacian regularization

Manifold regularization learning methods aim to explore the probability distribution of the geometric structure of data and punish the internal penalty function of the manifold as the regularization term itself. In manifold regularized SSL, based on fewer labeled data, the model learning performance with Laplacian may be dropped dramatically due to constant function in zero space [34]. Besides, high-order Hessian has a stronger learning ability of extrapolation using the richer zero space [35]. However, the prediction stability of Hessian can be affected significantly if the partial fitting function of the samples leads to significant shocks [36]. Compared with the above two manifold learning methods, *p*-Laplacian [37,38] is the nonlinear generalization of Laplacian and can better preserve the local geometric structure expression, using a more strict isoperimetric inequality, where the second eigenvector approximating the optimal Cheeger [39] reduces the arbitrarily well. In *p*-Laplacian regularization, the *p*-value is the key parameter to adjust the convergence of the graph *p*-Laplacian to the optimal Cheeger cut, where the *p*-value is equal to 2, the graph *p*-Laplacian turns to the standard graph Laplacian [19,20].

### 3.2 The proposed recognition method

Given a labeled and an unlabeled example set $L={\left\{x_{i}^{1},x_{i}^{2},\cdots ,x_{i}^{V},y_{i}\right\}}_{i=1}^{l}$ and $U={\left\{x_{i}^{1},x_{i}^{2},\cdots ,x_{i}^{V}\right\}}_{i=l+1}^{l+u}$, where *l* and *u* represent the cardinality of the labeled and unlabeled examples, respectively, *x*_*i*_ represents the *i*^th^ example, $x_{i}^{k}$, k∈{1,2,⋯,V} is the *k*^th^ information source eigenvector of the *i*^th^ example, *V* represents the number of sources, and $y_{i}\in \left\{-1,1\right\}$ is the category label of the *i*^th^ example. In general, *l ≪ u*. For SSL, the labeled examples are extracted from the probability distribution P, and the unlabeled examples are from the marginal distribution *P*_*x*_ of P, where *P*_*x*_ is a compact manifold M. Moreover, in *M,* the conditional distribution varies smoothly. Therefore, close example pairs have comparable conditional distribution pairs.

For convenience, the important notations are listed in Table 1.

**Table 1:** List of important notations.

Notation	Description	Notation	Description
*l*	Number of labeled samples	*u*	Number of unlabeled samples
***L***	Labeled sample set	***U***	Unlabeled sample set
*V*	Number of views	***x***^***k***^	The *k*^th^ view feature vector
*x*_*i*_	The *i*^th^ sample	$x_{i}^{k}$	The *k*^th^ view feature vector of the *i*^th^ sample
*y*_*i*_	The class label of the *i*^th^ sample	*f*	Classifier
${\left\|f\right\|}_{k}^{2}$	Classifier complexity penalty term	${\left\|f\right\|}_{I}^{2}$	Manifold classifier penalty term
$\gamma_{k}$	Parameter of ${\left\|f\right\|}_{k}^{2}$	$\gamma_{I}$	Parameter of ${\left\|f\right\|}_{I}^{2}$
*K*^*k*^	The *k*^th^ view kernel	*θ*^*k*^	Weight of the *k*^th^ view kernel
$Lp^{j}$	The *j*^th^ view *Lp*	$\beta^{j}$	Weight of the *j*^th^ view *Lp*
***K***	Multi-view kernel matrix	***Lp***	Multi-view *Lp* matrix
$\gamma_{\theta }$	Parameter of θ	$\gamma_{\beta }$	Parameter of β
**f**	Predicted vector of training samples	*α*_*i*_	The weight coefficient of the kernel function *K*(*x*, *x*_*i*_)
***H***_*k*_	Reproducing kernel Hilbert space (RKHS)		

The SSL classification problem is the optimization problem in (1) when considering an extra *p*-Laplacian regularization term to utilize the intrinsic geometry.

(1) $$\min_{f\in H_{k}}\frac{1}{l}\sum_{i=1}^{l}\psi \left(f,x_{i},y_{i}\right)+\gamma_{k}\;{||f||}_{k}^{2}+\gamma_{I}{||f||}_{I}^{2}$$

where ***H***_*k*_ represents an appropriate reproducing kernel Hilbert space (RKHS), ψ represents the loss function, ${||f||}_{k}^{2}$ is the classifier complexity penalization term in ***H***_*k*_, ${||f||}_{I}^{2}$ is the manifold regularization term for penalizing the classifier function *f* along the underlying manifold, and $\gamma_{k}$ and $\gamma_{I}$ are balancing parameters for the regularization terms ${||f||}_{k}^{2}$ and ${||f||}_{I}^{2}$ respectively.

The developed scheme combines graph *p*-Laplacian and multi-source kernel learning. Let ***K***^*k*^, k=1,2,⋯,V be a symmetric, positive definite kernel on the *k*^th^ source, and *θ*^*k*^ is a weight coefficient on the *k*^th^ source. The multi-source kernel is defined in (2) as follows:

(2) $$K=\sum_{k=1}^{V}\theta^{k}K^{k},s.t.\sum_{k=1}^{V}\theta^{k}=1,\theta^{k}\geq 0,k=1,2,\cdots ,V$$

The multi-source kernel ***K*** is a valid kernel. Thus the regularization term can be given as ${||f||}_{k}^{2}$ = **f**^***T***^***K*f** = $\sum_{k=1}^{V}\theta^{k}{||f||}_{K(k)}^{2}$, **f** is an estimated vector.

Equally, we define the multi-source *p*-Laplacian. Suppose ${L_{p}}^{j},\;j=1,2,\cdots ,V$ is the *p*-Laplacian of the *j*^th^ source, and *β* ^*j*^ is the weight coefficient of the *j*^th^ source. Then, we obtain (3) as follows:

(3) $$L_{p}=\sum_{j=1}^{V}\beta^{j}{L_{p}}^{j},\;s.t.\sum_{j=1}^{V}\beta^{j}=1,\beta^{j}\geq 0,\;j=1,2,\cdots ,V$$

where ${L_{p}}^{j}$ and ***L***_***p***_ are semi-definite positives. Then the regularization term can be obtained as ${||f||}_{I}^{2}$ = **f**^***T***^***L***_***p***_**f** = $\sum_{j=1}^{V}\beta^{j}{||f||}_{I(j)}^{2}$.

By introducing the regularization terms from (2), (3), (1), the optimization problem of (1) can be rewritten in (4) as follows:

(4) $$\min_{f\in H_{k},\theta \in R^{v},\beta \in R^{v}}\frac{1}{l}\sum_{i=1}^{l}\psi \left(f,x_{i},y_{i}\right)+\gamma_{k}\sum_{k=1}^{V}\theta^{k}{||f||}_{K(k)}^{2}+\gamma_{I}\sum_{j=1}^{V}\beta^{j}\;{||f||}_{I(j)}^{2}+\gamma_{\theta }{||\theta ||}_{2}^{2}+\gamma_{\beta }\;{||\beta ||}_{2}^{2}$$

$$\begin{array}{r} s.t.\sum_{k=1}^{V}\theta^{k}=1,\theta^{k}\geq 0,k=1,2,\cdots ,V\\ \;\sum_{j=1}^{V}\beta^{j}=1,\beta^{j}\geq 0,j=1,2,\cdots ,V \end{array}$$

where ${||\theta ||}_{2}^{2}$ and ${||\beta ||}_{2}^{2}$ are the regularization terms, $\gamma_{\theta },\gamma_{\beta }\in R^{+}$.

According to the representer theorem [40] and the given convex loss function, the optimization problem (4) becomes problem (5):

(5) $$f\left(x\right)=\sum_{i=1}^{l+u}\alpha_{i}K\left(x,x_{i}\right)$$

where *α*_*i*_ is the weight coefficient of the kernel function *K*(*x*, *x*_*i*_*)*.

By introducing (5) and the logistic loss function $\log\left(1+e^{-f}\right)$ into (4), optimizing the multi-source kernel logistic regression SSL and the *p*-Laplacian regularization becomes problem (6):

(6) $$\min_{f\in H_{k},\theta \in R^{v},\beta \in R^{v}}\frac{1}{l}\sum_{i=1}^{l}\left(\log\left(1+e^{-y_{i}K(x,x_{i})\alpha }\right)\right)+\gamma_{k}\;\alpha^{T}K\alpha +\gamma_{I}\alpha^{T}K\;L_{p}K\alpha +\gamma_{\theta }{||\theta ||}_{2}^{2}+\gamma_{\beta }{||\beta ||}_{2}^{2}$$

$$\begin{array}{r} s.t.\sum_{k=1}^{V}\theta^{k}=1,\theta^{k}\geq 0,k=1,2,\cdots ,V\\ \sum_{j=1}^{V}\beta^{j}=1,\beta^{j}\geq 0,j=1,2,\cdots ,V \end{array}$$

where $K=\sum_{k=1}^{V}\;\theta^{k}\;K^{k}$, $L_{p}=\sum_{j=1}^{V}\beta^{j}{L_{p}}^{j}$, ***α*** = [***α***_**1**_, ***α***_**2**_, …, ***α***_*l + u*_]^***T***^.

Next, we employ an alternating optimization [41] to solve (6) iteratively, where the first part fix θ and β to solve α, the second part fix α and β to solve θ, and the third part fix α and θ to solve β.

**The first part:** For a fixed θ and β, (6) becomes (7):

(7) $$\min_{\alpha \in R^{l+u}}\frac{1}{l}\sum_{i=1}^{l}\left(\log\left(1+e^{-y_{i}K(x,x_{i})\alpha }\right)\right)+\gamma_{k}\;\alpha^{T}K\alpha +\gamma_{I}\alpha^{T}K\;L_{p}K\alpha$$

where $K=\sum_{k=1}^{V}\;\theta^{k}\;K^{k}$ and $L_{p}=\sum_{j=1}^{V}\beta^{j}{L_{p}}^{j}$.

Based on the differentiable logistic loss function, we optimize (7) using the conjugate gradient algorithm. The first derivative of (7) can be written as:

$$\nabla f\left(\alpha \right)=-\frac{\log\;e}{l}\sum_{i=1}^{l}\left(\frac{y_{i}}{1+e^{y_{i}K(x,x_{i})\alpha }}\;K^{T}\left(x,x_{i}\right)\right)+\gamma_{k}\left(K+K^{T}\right)\alpha +\gamma_{I}\left(KL_{p}K+{\left(KL_{p}K\right)}^{T}\right)\alpha$$

Then, the solution is as follows:

Step1 Initialize $\alpha^{0}\;$, δ, $d^{0}=-\nabla f(a^{0})$, 0<ε≪1, m=0.

Step2 While $\left|f\left(\alpha^{m+1}\right)-f\left(\alpha^{m}\right)\right|>\varepsilon$, do

$$\alpha^{m+1}=\alpha^{m}+\delta d^{m}$$

$$d^{m+1}=-\nabla f(\alpha^{m+1})\;+\;\frac{{||\nabla f(\alpha^{m+1})||}^{2}}{{||\nabla f(\alpha^{m})||}^{2}}\;d^{m}$$

m=m+1

Step3 $\alpha^{*}=\alpha^{m+1}$.

**The second part:** For a fixed α and β, (6) becomes (8):

(8) $$\min_{\alpha \in R^{l+u}}\frac{1}{l}\sum_{i=1}^{l}\left(\log\left(1+e^{-y_{i}(\sum_{k=1}^{V}\;\theta^{k}\;K^{k}(x,x_{i}))\alpha }\right)\right)+\gamma_{k}\alpha^{T}\left(\sum_{k=1}^{V}\theta^{k}\;K^{k}\left(x,x_{i}\right)\right)\alpha \;+\gamma_{I}\alpha^{T}\left(\sum_{k=1}^{V}\theta^{k}\;K^{k}\left(x,x_{i}\right)\right)L_{p}\left(\sum_{k=1}^{V}\theta^{k}\;K^{k}\left(x,x_{i}\right)\right)\alpha +\gamma_{\theta }{||\theta ||}_{2}^{2}$$

$$s.t.\sum_{k=1}^{V}\theta^{k}=1,\theta^{k}\geq 0,k=1,2,\cdots ,V$$

where $L_{p}=\sum_{j=1}^{V}\beta^{j}{L_{p}}^{j}$.

The first derivative of (8) is:

$$\nabla f\left(\theta^{k}\right)=-\frac{\log\;e}{l}\sum_{i=1}^{l}(K^{k}\left(x,x_{i}\right)\alpha \frac{y_{i}}{1+e^{y_{i}\left(\sum_{k=1}^{V}\theta^{k}K^{k}\left(x,x_{i}\right)\right)\alpha }})+\gamma_{k}\alpha^{T}K^{k}\alpha \;+\gamma_{I}(2L_{p}{\left(\sum_{k=1}^{V}\theta^{k}K^{k}\left(x,x_{i}\right))\alpha \right)}^{T}K^{k}\alpha +\gamma_{\theta }\theta^{k}$$

Then we have the solution procedure presented below:

Step1 Initialize $\theta^{0}$, δ, 0<ε≪1, m=0, $d^{k^{\left(0\right)}}=-\nabla f(d^{k^{\left(0\right)}})$, k=1,2,⋯,V.

Step2 While $\left|f\left(\theta^{m+1}\right)-f\left(\theta^{m}\right)\right|>\varepsilon$, do

$$\theta^{k^{m+1}}=\theta^{k^{m}}+\delta d^{k^{m}}$$

$$d^{k^{m+1}}=-\nabla f(\theta^{k^{m+1}})+\frac{{||\nabla f(\theta^{k^{m+1}})||}^{2}}{{||\nabla f(\theta^{k^{m}})||}^{2}}\;d^{k^{m}}$$

m=m+1

Step3 $\theta^{*}=\theta^{m+1}$.

**The third part:** For a fixed α and θ, (6) becomes (9):

(9) $$\min_{\beta \in R^{l+u}}\;\gamma_{I}\;\alpha^{T}K\left(\sum_{j=1}^{V}\;\;\beta^{j}\;{L_{p}}^{j}\right)K\alpha +\;\gamma_{\beta }\;{||\beta ||}_{2}^{2}$$

$$s.t.\sum_{j=1}^{V}\beta^{j}=1,\beta^{j}\geq 0,j=1,2,\cdots ,V$$

where $K=\sum_{k=1}^{V}\;\theta^{k}\;K^{k}$.

Problem (9) is considered learning the optimal linear combination of multiple *p*-Laplacians.

The local-optimal solution of (6) is obtained by utilizing the alternating optimization algorithm.

### 3.3 Complexity analysis

Suppose we are given *n* examples, including *v* sources and *l* labeled samples. Let *k* be the iterations based on (7), (8). Then each time, optimizing α and θ becomes $ko\left(ln^{3}\right)$ and $ko\left(lvn^{3}\right)$, respectively. The time complexity of (9) is $o\left(v^{2}\right)$. Let η be the alternation iterations and γ be the number of the candidate parameter in *m* cross-validation. Then, our algorithm's time complexity is $o\left(m\eta \gamma \left(k\;ln^{3}+klvn^{3}+v^{2}\right)\right)$. Usually, *v* ≪ *n* and *k*, and therefore the time complexity is about $o\left(m\eta \gamma klvn^{3}\right)$.

## 4 The working condition recognition modeling

In this section, we first state the basis of selecting three feature information sources. Then the feature parameter extraction and calculation of each feature information source are described in detail through expert experience, prior knowledge and mechanism analysis based on theoretical dynamometer card. Finally, the modeling process of the working condition recognition based on the proposed method is introduced.

### 4.1 Feature information sources

The sucker-rod pumping system is an integrated complex nonlinear system. Hence, only comprehensive information analysis can be recognized promptly and accurately. Fig. 1 illustrates this comprehensive information extracted from the ground, wellbore, and stratum. Although the measured electrical power signals and ground dynamometer cards are ground-source information, the latter mainly reflects the condition from the wellbore and stratum, and the former is the condition from the ground and stratum. Although these information sources comprehensively reflect the working condition of the oil well, the similarity in the figure shape of the dynamometer card, the feature law of the electrical power card, and the wellhead information from the ground can only recognize a few working conditions accurately. For example, the wellhead temperature signal can precisely identify the working conditions between severe bottom leakage in tubing and assisted blowing. Moreover, the wellhead temperature increases assisted blowing and reduce leakage.

The existing technology allows massive multi-source information from oil and gas production to be measured and stored. So considering the above-existing limitations, we select the wellhead temperature signal, ground dynamometer cards, and electrical power signal as feature information sources.

### 4.2 Feature extraction

Given the feature extraction technology's limitations that rely on one information source to improve recognition and the engineering application, this work suggests a feature extraction scheme based on prior knowledge, expert experience, and mechanism analysis. It should be noted that this paper's feature information sources are extracted as a feature.

#### 4.2.1 Mechanism analysis based on theoretical dynamometer card

**Fig. 2:**
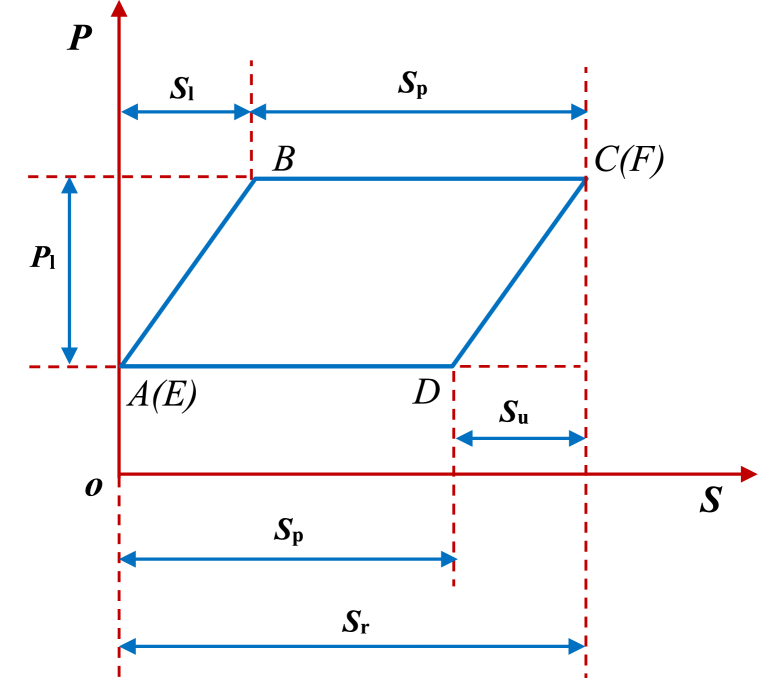
Theoretical dynamometer card.

The theoretical dynamometer card is drawn by the static load, while the polish rod is only affected by the sucker rod string and the liquid column on the piston. This is illustrated in Fig. 2, where the X-axis and Y-axis represent the displacement and load on the polish rod, denoted as S and P, respectively. Specifically, S_p_ is the piston stroke, i.e., the effective stroke, S_r_ is the polish rod stroke, P_l_ is the weight of the liquid column on the piston, S_u_ is the stroke loss of the unloading stage, and S_l_ is the stroke loss of the loading stage.

Besides, point *A(E)*, *B, C(F),* and *D* are the closing point of the traveling valve (i.e., the bottom dead center), the opening point of the standing valve, the closing point of the standing valve (i.e., the top dead center), and the opening point of the traveling valve. Thus, *A(E)*→*B*→*C(F)* is the upward stroke stage, involving the loading to the working process. Accordingly, *C(F)*→*D*→*A(E)* is the downward stroke stage, involving the unloading to the working process, and *A(E)*→*B*→*C(F)*→*D*→*A(E)* is a stroke, i.e., a working cycle time of oil well pump. The key to efficiently and accurately calculate the feature parameters of the above three measured information sources lies in the location to the valve opening and closing points (i.e., point *A(E)*, *B, C(F),* and *D*), and the four-point location adopts the technology based on prior knowledge, expert experience, and mechanism analysis [42] in this paper.

#### 4.2.2 Ground dynamometer card features

These features are extracted by changing the eight key factors in a stroke, including the key point position in the loading and unloading process, stroke loss, effective stroke, stroke, the weight of the liquid column on the piston, pump speed, load, and actual area of the dynamometer card. Moreover, we extract twelve features from the ground dynamometer cards, with the parameters and the corresponding measurement units presented in Table 2.

**Table 2:** The parameters and the corresponding measurement units of ground dynamometer card features, details on d_1_∼d_12_.

Parameter	notation	details
stroke (m), pump speed (min^−1^), maximal load (kN), minimal load (kN)	d_1_ (S_r_), d_2_, d_4_, d_5_	from the measured dynamometer card
area of the measured dynamometer card (kN·m)	d_3_	closed curve area of the measured dynamometer card
maximum and minimal load ratio	d_6_	the ratio between the maximum and the minimum loads
weight of liquid column on piston (kN)	d_7_ (P_l_)	difference between the maximum and the minimum load
effective stroke (m)	d_8_ (S_p_)	the opening and closing point displacement difference of the traveling valve
stroke loss of loading (m)	d_9_ (S_l_)	displacement difference between the opening point at the standing valve and the closing point at the traveling valve
stroke loss of unloading (m)	d_10_ (S_u_)	displacement difference between the closing point at the standing valve and the opening point at the traveling valve
advanced loading position (m)	d_11_	displacement of the first point (i.e., the advanced loading point) in the reverse slope's positive and negative direction from the closing to the opening point in the traveling valve
advanced unloading position (m)	d_12_	displacement of the first point (i.e., the advanced unloading point) in a reverse slope's positive and negative direction of the slope from the closing to the opening point in the standing valve

#### 4.2.3 Electrical power signal features

The electrical power signal's features are obtained using the “AUC (area under the curve)” and “power feature” [43]. Specifically, seven features are extracted from the electrical power signal, with the parameters and the corresponding measurement units presented in Table 3.

**Table 3:** The parameters and the corresponding measurement units of electrical power signal features, details on e_1_∼e_7_.

Parameter	Notation	Details
Uplink power (kW)	e_1_	Total power of the upward stroke stage
Downlink power (kW)	e_2_	Total power of the downward stroke stage
Period power (kW)	e_3_	Total uplink and the downlink power
Uplink area (kW·m)	e_4_	The area enclosed by the time-series horizontal axis and the electric power signal curve during the upward stroke stage
Downlink area (kW·m)	e_5_	The area enclosed by the time-series horizontal axis and the electric power signal curve during the downward stroke stage
Period area (kW·m)	e_6_	Total uplink and the downlink area
balance rate	e_7_	the ratio between the uplink power and the downlink power

#### 4.2.4 Wellhead temperature signal features

According to Ref. [21] and based on the mechanism analysis, the wellhead temperature signal features are extracted from the heat loss during a stroke. Specifically, three features are obtained from the wellhead temperature signal. The parameters and the corresponding measurement units are the uplink heat loss (°C), the downlink heat loss (°C), and the period heat loss (°C), expressed by t_1_∼t_3_, respectively. Among them, t_1_ and t_2_ are the total heat loss during the upward and downward stroke stages, respectively, and t_3_ is the total uplink and downlink heat loss.

### 4.3 Modeling process for the operating state recognition

**Fig. 3:**
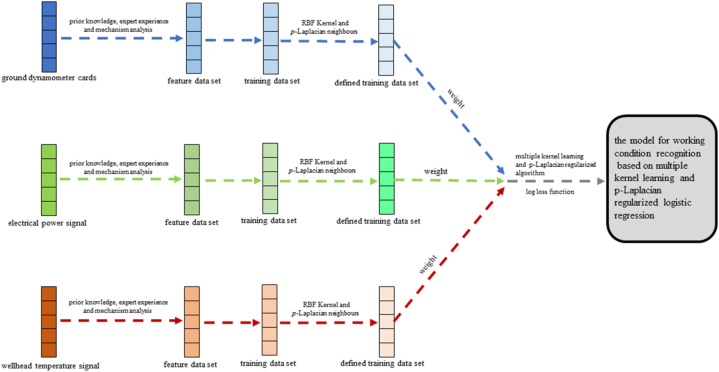
Operating condition recognition architecture for sucker-rod pumping wells using multi-source kernel learning and *p*-Laplacian regularized logistic regression.

The modeling process for the operating state recognition for sucker-rod pumping wells relying on multi-source kernel learning and *p*-Laplacian regularized logistic regression is illustrated in Fig. 3.

In this process, establishing the model for recognizing the operating conditions effectively requires meticulously selecting the kernel function, neighbors and the *p*-value of *p*-Laplacian, the weight of each source, model parameters ($\gamma_{k}$, $\gamma_{I}$, $\gamma_{\theta }$, $\gamma_{\beta }$), and iterations of alternating optimization solution. Thus, the RBF kernel function is chosen, the *p*-Laplacian neighbors equal the sample cardinality per category, the total weights per source are set to one, the iterations are 1200, the *p*-value of *p*-Laplacian and the model parameters are configured after tuning.

The working condition recognition scheme can be described as follows. First, the three proposed measured feature sources from sucker-rod pumping wells are obtained. Thus, the corresponding feature data sets of the operating conditions are established to generate the training and testing data. Once the kernel function, neighbors, the *p*-value of *p*-Laplacian, and the weight of each source are defined, the data set is trained using its kernel matrix ***K*** and *p*-Laplacian matrix ***L***_***p***_. Next, the proper model parameters are tuned based on the training data to obtain the optimum solution after 1200 iterations.

## 5 Experiment and discussion

The experimental data are collected from 60 sucker-rod pumping wells in a block of a certain oil field in China. The block reservoir type is a typical thin oil reservoir with high pressure and low permeability. The operating condition samples are produced strictly from the working record of the oil wells, and the operation state data set is established through the collected data derived from three proposed feature sources during the same period. The data set comprises triennial operating condition data, including 11 types of typical operating conditions (normal, rod cutting, assist-blowing, lack of supply liquid, traveling valve failing, stuck pump, wax precipitation, pump leakage, tubing leakage, standing valve leakage, and traveling valve leakage), with 150 samples per type and 1650 samples in total.

The experiments are conducted on the matlab R2016b numerical analysis software and win 7.0 SP1/2 GB NVIDIA GeForce820 M GPU operating system.

In the following experiments, the operating condition data set is uniformly divided into the training and the testing set, each set having 825 samples, with 75 of each kind. The parameter $\gamma_{k}$ is tuned using the candidate set $\left\{10^{e}|e=-5,-4,-3,-2,-1,0\right\}$, $\gamma_{I}$ is tuned using the candidate set $\left\{10^{e}|e=-4,-3,-2,-1,0\right\}$, and $\gamma_{\theta }$ and $\gamma_{\beta }$ are tuned using the candidate set $\left\{10^{e}|e=-2,-1,0\right\}$, the *p*-value of *p*-Laplacian is tuned using the candidate set {1,1.01,1.02,...,3}. Additionally, the parameters *θ* and *β* are tuned based on the equation $\theta^{1}+\theta^{2}+\theta^{3}=\beta^{1}+\beta^{2}+\beta^{3}=1$. The kernel function is set to RBF, and the neighbors of *p*-Laplacian are set to 75, and the *p*-value of *p*-Laplacian after debugging is 1.92. The binary classifier is used, and the maximum iterations are 1200.

Our experiments have three aims. First, to evaluate our method's effectiveness and challenge it against current recognition methods based on labeled training samples. Second, evaluate our method's practicability and challenge it against existing recognition methods using differently labeled training samples. Third, evaluate the robustness of the proposed method and compare the results between the *p*-Laplacian and Hessian manifold learning based on very few labeled samples and two significantly different neighbors.

### 5.1 Evaluating various recognition methods schemes utilizing all labeled training samples

The developed multi-source kernel learning and *p*-Laplacian regularized logistic regression recognition method (mL_p_LR) is challenged against the single information source recognition method (LR), *p*-Laplacian regularized single information source recognition method (L_p_LR), and the feature connection multiple information source recognition method (mCLR). The experimental results are reported in Table 4, highlighting that in the scenario using labeled training samples, mL_p_LR attains a higher recognition performance than LR and L_p_LR. Moreover, compared to recognition methods based on ground dynamometer cards, mL_p_LR improves recognition by about 2.4% and 2.5% relative to LR and L_p_LR. Regarding the recognition schemes utilizing the electric power signal, mL_p_LR enhances recognition by about 11% relative to LR and L_p_LR.

**Table 4:** Comparison results of different recognition methods using labeled training samples.

Source N	Information Source	LR (%)	L_p_LR (%)	mCLR (%)	mL_p_LR (%)
1	Ground dynamometer cards	94.84	94.70		
1	electric power signal	86.20	86.20		
2	Electric power signal and ground dynamometer			84.98	**96.77**
3	Wellhead temperature signal, electric power signal, and ground dynamometer cards			83.47	**97.23**

Meanwhile, mL_p_LR achieves a much better recognition performance than traditional mCLR. Besides, regarding the recognition schemes utilizing ground dynamometer cards and electric power signals, mL_p_LR improves the recognition rate by about 11.8%. Furthermore, recognition methods based on wellhead temperature signals, electric power signals, and ground dynamometer cards, mL_p_LR, enhance the recognition rate by 13.8%. Table 4 indicates that mL_p_LR can improve recognition accuracy by exploiting new feature data, while mCLR has the opposite effect. In the experiments, the single source and *p*-Laplacian regularization-based methods have a minor effect on the recognition rate, but methods relying on multiple sources and *p*-Laplacian regularization can enhance the recognition rate.

Overall, for labeled training samples, except for mCLR, all recognition methods obtain better recognition results using different sources, including the single source based on ground dynamometer cards, the combined sources based on electric power signal and ground dynamometer cards, and the combined sources based on wellhead temperature signal, electric power signal, and ground dynamometer cards.

### 5.2 Evaluating various recognition methods utilizing different labeled training samples

In the subsequent trials, we consider the recognition algorithms that attained the highest performance in the previous experiments. Meanwhile, the class-aware contrastive semi-supervised deep learning method (CCSSL) is also added. The experiments based on deep learning are conducted on python 3.8.10/pytorch 1.10.0 and linux 5.4.0/24 GB NVIDIA RTX3090 GPU. Considering the recognition effect of one-dimensional discrete feature data in CCSSL, the experimental data based on CCSSL only selects ground dynamometer cards. The data set samples and the data set division are the same as the previous experiments, but the original binary ground dynamometer card data (200*200 pixels) is selected for the feature data. Hence, we challenge mL_p_LR against LR, L_p_LR and CCSSL under five groups of different labeled training samples (i.e. 10%, 30%, 50%, 70%, 100% five groups of ratios, corresponding to 8, 23, 38, 53, 75 labeled samples in each kind of working condition). Table 5 reports the corresponding experimental results and reveals that it is challenging to obtain working condition samples due to the complex and variable working conditions. However, the sucker-rod pumping system can obtain massive unknown working condition samples in big data oil production. Compared with the competitor schemes, the suggested mL_p_LR algorithm affords a more precise recognition effect utilizing the right amount of labeled samples and many unlabeled samples. Hence, our method is more suitable for engineering applications.

**Table 5:** Comparison results of different recognition methods based on different labeled training samples.

Source N	Information Source	Different methods	10% labeled (%)	30% labeled (%)	50% labeled (%)	70% labeled (%)	100% labeled (%)
1	Ground dynamometer cards	LR	84.63	87.73	90.40	93.17	94.84
1	Ground dynamometer cards	L_p_LR	84.57	87.53	89.98	93.50	94.70
1	Ground dynamometer cards (binary image)	CCSSL	88.44	91.81	92.10	91.45	92.15
3	Wellhead temperature signal, electric power signal, and ground dynamometer cards	**mL**_**p**_**LR**	**87.55**	**90.86**	**92.54**	**94.97**	**97.23**

The suggested mL_p_LR scheme affords better recognition than the single source recognition methods LR and L_p_LR when employing different labeled training samples. Specifically, for 30% of labeled samples, the recognition precision improves by about 3%. For less than 30% of labeled samples, mL_p_LR is still better than LR and L_p_LR. Compared with CCSSL based on automatic feature learning, the recognition precision of mL_p_LR is slightly lower than that of CCSSL in 30% or less of labeled samples. However, overall, mL_p_LR has better recognition effect in different labeled samples. It shows that the proposed feature extraction method and *p*-Laplacian high-order manifold data structure can better improve the semi-supervised learning method and model performance.

### 5.3 Comparison results between *p*-Laplacian and Hessian manifold learning based on very few labeled samples and two larger differences in neighbor numbers

**Fig. 4:**
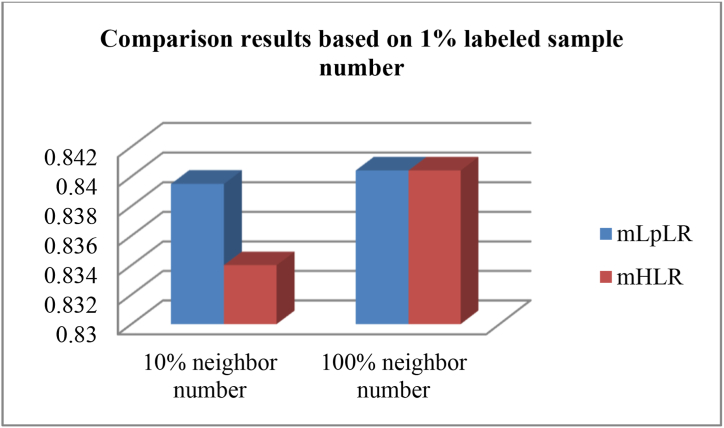
Comparison results between mL_p_LR and mHLR based on 10% and 100% neighbor sample number of each category in the case of **1%** labeled sample number of each category.

Next, we select two multi-source kernel recognition methods based on high-order manifold learning. These are the multi-source kernel learning, *p*-Laplacian regularized logistic regression recognition method (mL_p_LR), multi-source kernel learning, and Hessian regularized logistic regression recognition method (mHLR) [44]. The following experiment considers recognizing the working condition for 1% labeled samples (i.e. 1 labeled sample) per category, combined with 10% (i.e. 8 samples) and 100% (i.e. 75 samples) neighbor samples per category. Essentially, we select 1% labeled samples to identify the working condition, i.e., we select one labeled sample and 74 unlabeled samples. In this case, the data are prone to severe swings, significantly affecting the model's stability and the recognition effect. The corresponding experimental results are reported in Fig. 4, demonstrating that mHLR is slightly inferior to mLpLR for a 10% neighbor sample number. However, both methods afford the same recognition effect for a 100% neighbor sample number and attain better recognition than the schemes relying on a 10% neighbor sample number. Overall, the experimental results reveal that mLpLR is quite robust.

## 6 Conclusion

In order to address limitations in the current operating state recognition research of sucker-rod pumping system and further improve the recognition effect, engineering practicability and model robustness of operation state recognition methods in the case of big data oil recovery production, this paper develops an operating state recognition scheme utilizing multi-source nonlinear kernel learning and *p*-Laplacian manifold learning. Indeed, aiming at the recognition limitation of single information source in electron machinery liquid integrating complex nonlinear system and the technology bottleneck of traditional multi-feature connection learning, the multiple nonlinear kernel learning algorithm based on multiple measured information sources is proposed to further improve the operating state recognition effect. Meanwhile, in order to solve the engineering practicability problem caused by the demand of a large number of labeled samples and the model robustness problem triggered by small number of labeled samples, the *p*-Laplacian regularization is introduced into the multi-source kernel SSL with log loss function to enhance engineering practicability and model robustness by fewer labeled and massive unlabeled samples. Furthermore, in view of the effect on the operating state recognition modeling by the accuracy, efficiency and practicality of the feature parameter calculation, the feature extraction method based on prior knowledge, expert experience, and mechanism analysis is proposed to further heighten model effectiveness. The ground dynamometer cards, electrical power signal, and wellhead temperature signal are selected and extracted as feature information sources by the above proposed method. The operating state recognition model is implemented by the three measured information sources, and it can be good for improving the model performance. Extensive experiments considering fully labeled and the right amount of labeled sample (above 30%) scenarios demonstrate that our scheme has a better recognition effect than the current popular class-aware contrastive semi-supervised deep learning algorithm based on the binary ground dynamometer cards and classic algorithms relying on a single-source data set and multiple feature connection data set based on the proposed feature extraction method.

In this paper, a general operating state recognition approach of sucker-rod pumping system is proposed. However, there are still some additional studies to be further developed. Attempt to combine multiple information sources and intelligent optimization algorithms with deep learning to develop a more efficient, robust and practical operating state recognition model in very small amount of labeled sample scenarios. In addition, try to develop an effective and active learning semi-supervised operating state recognition model to challenge complex and variable working conditions.

## Ethics declarations

1. Review and/or approval by an ethics committee was not needed for this study because [Our study is a managerial empirical study and does not involve any ethical issues.].
2. Informed consent was not required for this study because [The data for this study was informed for academic purposes and did not involve any issues requiring informed consent.].

## Funding

This research was funded by the 10.13039/501100007129Shandong Provincial Natural Science Foundation of China [grant number ZR2021MF031].

## Data availability statement

Data will be made available on request.

## CRediT authorship contribution statement

**Bin Zhou:** Writing – original draft, Supervision, Methodology, Funding acquisition, Conceptualization. **Rui Niu:** Writing – review & editing, Writing – original draft, Supervision, Methodology, Conceptualization. **Shuo Yang:** Validation, Methodology, Investigation, Data curation. **Jianguo Yang:** Writing – review & editing, Validation, Software, Formal analysis, Data curation. **Weiwei Zhao:** Writing – review & editing, Validation, Software, Formal analysis, Data curation.

## Declaration of competing interest

The authors declare that they have no known competing financial interests or personal relationships that could have appeared to influence the work reported in this paper.
